# Supplementary material for: Time-series transcriptome analysis identified differentially expressed genes in broiler chicken infected with mixed Eimeria species
Source: Front Genet. 2022 Aug 8;13:886781. doi: 10.3389/fgene.2022.886781 (PMC9393255; doi:10.3389/fgene.2022.886781)
Supplement: Supplementary file 2 [file DataSheet1.ZIP › 7dpi_GO.Gsea.1625106495923/GOBP_BLASTODERM_SEGMENTATION.html]

Details for gene set GOBP\_BLASTODERM\_SEGMENTATION[GSEA]

|  || Dataset | TMM\_7dpi\_gct\_format\_7dpi\_gct\_format.Class\_4dpi.cls #PC\_versus\_NC.Class\_4dpi.cls #PC\_versus\_NC\_repos |
| Phenotype | Class\_4dpi.cls#PC\_versus\_NC\_repos |
| Upregulated in class | 0 |
| GeneSet | GOBP\_BLASTODERM\_SEGMENTATION |
| Enrichment Score (ES) | -0.75190806 |
| Normalized Enrichment Score (NES) | -1.805753 |
| Nominal p-value | 0.0010905125 |
| FDR q-value | 0.12069741 |
| FWER p-Value | 0.9528 |
Table: GSEA Results Summary

  

Fig 1: Enrichment plot: GOBP\_BLASTODERM\_SEGMENTATION      
 Profile of the Running ES Score & Positions of GeneSet Members on the Rank Ordered List

  

| SYMBOL | TITLE | RANK IN GENE LIST | RANK METRIC SCORE | RUNNING ES | CORE ENRICHMENT || 1 | WNT5A | na | 2943 | 0.133 | -0.2270 | No |
| 2 | WDR77 | na | 4212 | -0.019 | -0.3302 | No |
| 3 | SEMA3A | na | 4416 | -0.041 | -0.3414 | No |
| 4 | NRP2 | na | 5691 | -0.168 | -0.4246 | No |
| 5 | SEMA3F | na | 7273 | -0.337 | -0.5103 | No |
| 6 | HOXD8 | na | 7354 | -0.347 | -0.4696 | No |
| 7 | PLD6 | na | 9272 | -0.594 | -0.5482 | No |
| 8 | FRS2 | na | 11717 | -1.256 | -0.5803 | Yes |
| 9 | TBX3 | na | 11786 | -1.314 | -0.4066 | Yes |
| 10 | BASP1 | na | 11851 | -1.421 | -0.2180 | Yes |
| 11 | TBXT | na | 11959 | -1.693 | 0.0042 | Yes |
Table: GSEA details [plain text format]

  

Fig 2: GOBP\_BLASTODERM\_SEGMENTATION      
 Blue-Pink O' Gram in the Space of the Analyzed GeneSet

  

Fig 3: GOBP\_BLASTODERM\_SEGMENTATION: Random ES distribution      
 Gene set null distribution of ES for **GOBP\_BLASTODERM\_SEGMENTATION**
